# Epidemiological, clinical and microbiological aspects of infective endocarditis in Türkiye

**DOI:** 10.1007/s10096-025-05095-8

**Published:** 2025-03-14

**Authors:** Elif M. Saricaoglu, Seniha Basaran, Derya Seyman, Merve Arslan, Serpil Ozkan-Ozturk, Yasemin Tezer-Tekce, Yesim Uygun-Kizmaz, Nuran Sari, Denef Berzeg-Deniz, Alpay Azap, Serap Simsek-Yavuz, Ozlem Kurt-Azap, Türkiye Endocarditis Group (TEG) Investigators

**Affiliations:** 1 Department of Infectious Disease and Clinical Microbiology, Ankara University Faculty of Medicine, Ankara, Turkey; 2 Department of Infectious Disease and Clinical Microbiology, Istanbul Faculty of Medicine, Istanbul University, Istanbul, Turkey; 3 Department of Infectious Disease and Clinical Microbiology, Antalya Training and Research Hospital, Antalya, Turkey; 4 Department of Infectious Disease and Clinical Microbiology, Pamukkale University Faculty of Medicine, Denizli, Turkey; 5 Department of Infectious Disease and Clinical Microbiology, Mehmet Akif Ersoy Thoracic and Cardiovascular Surgery Training and Research Hospital, Istanbul, Turkey; 6 Department of Infectious Disease and Clinical Microbiology, Ankara Bilkent City Hospital, Ankara, Turkey; 7 Department of Infectious Disease and Clinical Microbiology, Kosuyolu Yuksek Ihtisas Training and Research Hospital, Istanbul, Turkey; 8 Department of Infectious Disease and Clinical Microbiology, Ankara Baskent University Faculty of Medicine, Ankara, Turkey; 9 Department of Infectious Disease and Clinical Microbiology, Dr. Siyami Ersek Thoracic And Cardiovascular Surgery Training and Research Hospital, Istanbul, Turkey

**Keywords:** Epidemiology, Infective endocarditis, Mortality, Türkiye

## Abstract

**Purpose:**

Infective endocarditis (IE) is a evolving disease with a shifting epidemiology and disease burden over time. This study aimed to compare the epidemiological and clinical aspects of IE over three time periods across eleven years.

**Methods:**

This was a retrospective cohort, multicenter study conducted in Türkiye, comparing three periods: 2013–2016, 2017–2020, and 2021–2023. Epidemiological and microbiological characteristics, as well as patient outcomes, were analyzed and compared across these periods.

**Results:**

A total of 1,044 patients diagnosed with IE were included. The median (Q1-Q3) age was 57 (44–68) years, with an increasing pattern (*p* < 0.001). Throughout the study period, the prevalence of intracardiac devices increased, whereas the prevalence of degenerative and congenital heart diseases declined. Among all patients, the most frequently identified pathogens were staphylococci (36.4%), followed by streptococci (14.0%) and enterococci (11.9%). Throughout the three periods, there was a significant increase in staphylococci, with *S. aureus* emerging as the predominant pathogen in all type IE. The in-hospital mortality rate among all patients was 22.5%. Independent risk factors for in-hospital mortality included ≥ 65 age(OR = 1.9), chronic kidney disease (OR = 1.9), nosocomial acquisition (OR = 2.1), *Candida* spp. infection (OR = 2.9), prosthetic valve IE (OR = 1.9), vegetation size > 15 mm (OR = 1.6), and central nervous system emboli (OR = 2).

**Conclusion:**

The epidemiology of IE is undergoing significant changes, leading to shifts in microbiological profiles and clinical presentations. Effective management of IE should be guided by established clinical guidelines while integrating up-to-date epidemiological data to ensure comprehensive and evidence-based patient care.

**Supplementary Information:**

The online version contains supplementary material available at 10.1007/s10096-025-05095-8.

## Introduction

Infective endocarditis (IE) is an evolving disease characterized by an increasing burden and significant changes in its epidemiology, microbiology, clinical presentation, and outcomes over time [1]. Recent studies have reported that the annual incidence of infective endocarditis ranges from 2.6 to 13.8 cases per 100,000 people [2, 3, 4]. Over the past 30 years, the incidence of IE has significantly increased across all age groups [2]. Notable epidemiological shifts in the 21st century include the decline of rheumatic heart disease, which has been proportionally replaced by degenerative valvulopathies, prosthetic valves, and intracardiac devices (ICDs) [5]. Additionally, the median age of IE patients has risen significantly [6].

In recent years, *Staphylococcus aureus* has emerged as the most common pathogen in IE patients. While *Streptococcus* species are encountered less frequently, they remain prevalent in low-income countries where rheumatic valvular disease is still a major risk factor. A substantial proportion of community-acquired, nosocomial, and non-nosocomial healthcare-associated (HCA-IE) cases are associated with *S. aureus* [7]. Due to the predominance of *S. aureus*, particularly in patients with multiple comorbidities, IE is increasingly described as having an acute disease course [8]. Despite significant advances in diagnosis and treatment, mortality rates in European regions have remained relatively stable, ranging between 18% and 21%, due to the increasing number of complicated cases [7]. However, this increasing clinical complexity has contributed to persistently high mortality rates over the past few decades [5]. In Türkiye, a study by Şimşek-Yavuz et al. reported a hospital mortality rate of 27.8% for IE [9].

Given the evolving epidemiology and outcomes of IE, understanding these changes is crucial for improving patient management. The aim of this study was to assess the epidemiological aspects and outcomes of IE over three distinct time periods spanning eleven years.

## Material-methods

The Türkiye Endocarditis Group (TEG) cohort is a national, multicenter registry from 2013 that has included a total of 40 centers across Türkiye. This study is a retrospective observational analysis based on the collected data. The primary centers for the diagnosis and treatment of infective endocarditis in our country remained constant throughout this study period, while additional centers were incorporated into the study cohort over time. A total of 1,044 patients with definite or possible IE, based on the Modified Duke Criteria [10], were enrolled in this cohort between January 2013 and December 2023 from 12 geographic regions of Türkiye, classified according to the Nomenclature of Territorial Units for Statistics-1 (NUTS-1). Only the first episode of IE for each patient was considered.

The data analyzed included demographic information, comorbidities, predisposing conditions, type of IE acquisition, type of endocarditis, echocardiographic findings, causative microorganisms, clinical manifestations, complications, and outcomes. Detailed variables are presented in Table 1 and Supplement 1. Three time periods were evaluated: 2013–2016, 2017–2020, and 2021–2023. Comparisons across these periods focused on epidemiology, microbiology, clinical features, and outcomes.

The following analytical strategies were employed:

- Changes in the epidemiological, microbiological, and clinical characteristics of IE were analyzed across the three time periods.
- A comparison between survivors and non-survivors was conducted to identify mortality risk factors.

IE was classified according to the 2015 European Society of Cardiology (ESC) guideline [10], which categorize IE into possible or definite IE; native valve IE, prosthetic valve IE, and cardiac implantable electronic device-associated IE (CIED-IE). The classification for the type of acquisition was based on the definitions previously described by Benito et al. [11], follows as; (a) community-acquired IE, (b) nosocomial IE, and (c) non-nosocomial HCA-IE. Complications were identified using diagnostic imaging, including cerebral, splenic, and pulmonary embolisms, as well as spondylodiscitis.

### Statistical analysis

The distribution of variables was assessed using visual methods (histograms, probability plots) and analytical methods (Kolmogorov-Smirnov/Shapiro-Wilk tests) to determine normal distrubition. Descriptive statistics were reported as medians and interquartile range (IQR) for non-normally distributed and ordinal variables, and frequencies for categorical variables. Kruskal-Wallis tests were conducted to compare non-normally distributed and ordinal variables. The Mann-Whitney U test was performed to test the significance of pairwise differences, with Bonferroni correction applied for multiple comparisons. An overall 95% type-I error level was used to determine statistical significance. Fisher’s exact test or the Chi-square test, where appropriate, was used to compare categorical variables between groups. Variables associated with in-hospital mortality were first evaluated using univariate analysis. Variables with a p-value of < 0.05 were then included in a multivariate logistic regression analysis, based on clinical relevance, to identify independent risk factors. All statistical analyses were performed using SPSS software (Version 23, IBM SPSS, Armonk, NY, USA).

This study was performed in line with the principles of the Declaration of Helsinki. This study was approved by the Ethics Committee of the Faculty of Medicine at Ankara University on March 28, 2024, with Decision Number: İ03-239-24.

## Results

A total of 1,044 episodes of IE were included in the study. 223 cases (21.4%) were classified as possible IE, while 821 cases (78.6%) were classified as definite IE. There were 205 cases in the first period (2013–2016), 491 cases in the second period (2017–2020), and 348 cases in the last period (2021–2023). The median age of patients was 57 years (Q1–Q3 = 44–68), showing an increasing pattern from 54 to 60 years over the study period (*p* < 0.001). The majority of the cohort were male (*n* = 666, 63.8%). Native valve IE, prosthetic valve IE, and CIED-IE were diagnosed in 659 (63%), 307 (29.4%), and 78 (7.5%) of patients, respectively.

The most common comorbidities were hypertension (HT) (*n* = 382, 36.6%), diabetes mellitus (DM) (*n* = 282, 27%), and CKD (*n* = 220, 21.1%), with a significant increase observed in the rates of HT (*p* < 0.001), DM (*p* < 0.001), and malignancy (*p* = 0.015) over the study period. The most frequently identified predisposing risk factors included prosthetic valves (*n* = 323, 30.9%), degenerative valvular disease (*n* = 183, 17.5%), hemodialysis (HD) (*n* = 169, 16.2%), and ICDs (*n* = 102, 9.8%). In contrast, the percentage of patients with rheumatic fever and congenital heart disease (CHD) decreased from 11.2 to 7.2% and from 8.3 to 3.2%, respectively. The proportion of intravenous drug users (IVDU) in this cohort was 4.5% (Table 1).

**Table 1:** Epidemiological and clinical characteristics for infective endocarditis cohort stratified by three time periods

	Total (*n* = 1044)	2013–2016 (*n* = 205)	2017–2020 (*n* = 491)	2021–2023 (*n* = 348)	*p* value	Post-hoc analysis
**Demographics**						
Age in years	57(44,68)	54 (40,65)	55(43,66)	60(47,70)	**< 0.001**	**1 = 2**,** 2 < 3**
18–39 y 40–64 y ≥65 y	202(19.3) 517(49.6) 325(31.1)	48(23.4) 104(50.7) 53(25.9)	98(20) 256(52.1) 137(27.9)	56(16.1) 157(45.1) 135(38.8)	**< 0.001**	**1**,**2 < 3**
Male sex	666(63.8)	133(64.9)	310(63.1)	223(64.1)	0.901	
**Comorbidities**						
Hypertension	382(36.6)	66(32.2)	157(32)	159(45.7)	**< 0.001**	**1 = 2**,** 2 < 3**
Diabetes mellitus	282(27)	39(19)	114(23.2)	129(37.1)	**< 0.001**	**1**,**2 < 3**
Chronic kidney disease	220(21.1)	40(19.5)	97(19.8)	83(23.9)	0.297	
Malignancy	57(5.5)	9(4.4)	19(3.9)	29(8.3)	**0.015**	**1 = 2**,** 2 < 3**
Autoimmune disease	44(4.2)	3(1.5)	23(4.7)	18(5.2)	0.086	
**Predisposition conditions**						
Prosthetic valve	323(30.9)	59(28.8)	166(33.8)	98(28.2)	0.166	
Degenerative heart disease	183(17.5)	48(23.4)	89(18.1)	46(13.2)	**0.009**	**1**,**2 > 3**
Hemodialysis	169(16.2)	27(13.2)	84(17.1)	58(16.7)	0.419	
Intracardiac device	102(9.8)	15(7.3)	39(7.9)	48(13.8)	**< 0.001**	**1 = 2 < 3**
Rheumatic fever	87(8.3)	23(11.2)	39(7.9)	25(7.2)	0.060	
Bicuspid aortic valve	64(6.1)	16(7.8)	35(7.1)	13(3.7)	0.070	
Congenital heart disease	60(5.7)	17(8.3)	32(6.5)	11(3.2)	**0.026**	**1**,**2 > 3**
Previous IE episode	52(5)	14(6.8)	22(4.5)	16(4.6)	0.397	
Intravenous drug user	47(4.5)	6(2.9)	28(5.7)	13(3.7)	0.192	
**Type of acquisition**						
Community acquired	619(59.3)	133(64.9)	269(54.8)	217(62.4)	0.709	
Nosocomial	157(15)	29(14.1)	72(14.7)	56(16.1)	**0.010**	**1 = 2**,** 2 < 3**
Non-nosocomial healthcare-associated infection	268(25.7)	43(21)	150(30.5)	75(21.6)	0.484	
**Endocarditis side**						
Mitral	469(44.9)	90(43.9)	217(44.2)	162(46.6)	0.510	
Aortic	402(38.5)	102(49.8)	203(41.3)	97(27.9)	**< 0.001**	**1**,**2 > 3**
Tricuspid	111(10.6)	15(7.3)	51(10.4)	45(12.9)	**0.035**	**1**,**2 < 3**
Pulmonary	13(1,3)	3(1.5)	7(1.4)	3(0.1)	0.561	
Lead	70(6.7)	8(3.9)	28(5.7)	34(9.8)	**0.011**	**1**,**2 < 3**
Endocard	21(2)	4(2)	8(1.6)	9(2.6)	0.189	
**Type of IE**						
Native-valve IE	659(63.1)	136(66.4)	303(61.7)	221(63.2)	0.482	
Prosthetic-valve IE	307(29.4)	59(28.7)	156(31.8)	93(26.7)	0.266	
CIED-IE	78(7.5)	10(4.9)	32(6.5)	35(10.1)	**0.048**	**1 = 2**,** 2 < 3**
**Complications**						
Cranial embolism	203(19.4)	37(18)	106(21.6)	60(17.2)	0.250	
Splenic infarct/abcess	23(22.2)	2(1)	19(3.9)	2(0.6)	0.353	
Glomerulonephritis	29(2.8)	1(0.5)	21(4.3)	7(2)	0.593	
Pulmonary embolism	77(7.4)	2(1)	32(6.5)	43(12.4)	**< 0.001**	**1**,**2 < 3**
Spondylodiscitis	24(2.3)	1(0.5)	7(1.4)	16(4.6)	**0.002**	**1 = 2**,** 2 < 3**
Heart failure	120(11.5)	10(4.9)	52(10.6)	58(16.7)	**< 0.001**	**1**,**2 < 3**
**Cardiac surgery for IE**	406(38.8)	102(49.8)	196(39.9)	108(31.1)	**< 0.001**	**1**,**2 > 3**
**In-hospital mortality**	235(22.5)	38(18.5)	112(22.8)	85(24.4)	0.271	

**Fig. 1:**
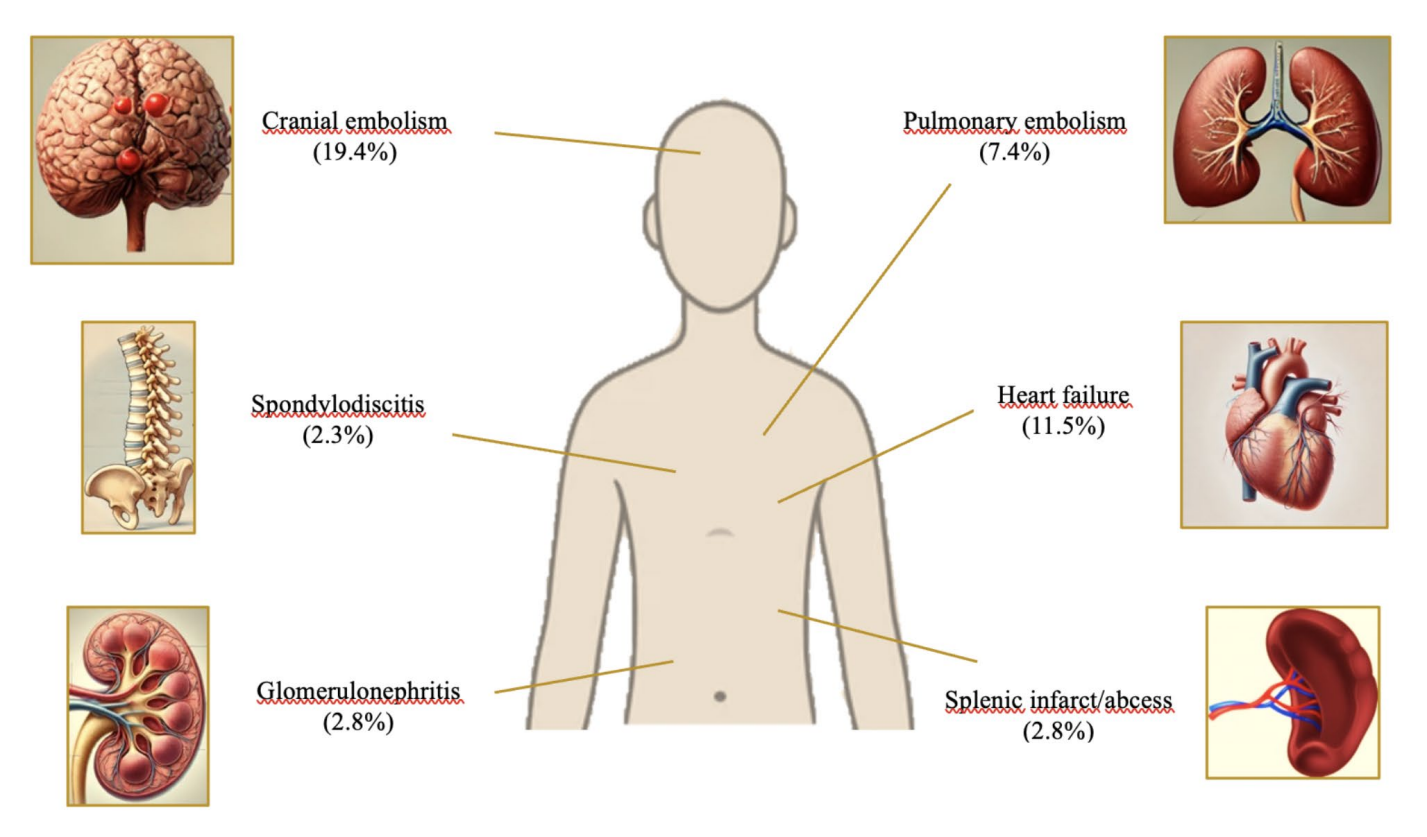
The percentages of infective endocarditis complications in the study cohort

Tricuspid valve and lead involvement, along with right-sided IE, demonstrated a statistically significant increase over the last decade (*p* = 0.035, *p* = 0.011). The study also revealed a significant increase in the prevalence of CIED-IE (*p* = 0.048). During this period, complications such as pulmonary embolism (*p* < 0.001), spondylodiscitis (*p* = 0.002), and heart failure (*p* < 0.001) became more frequent. The frequency of complications observed in the entire cohort was illustrated in Fig. 1. The rate of surgical interventions declined from 49.8 to 31.1% (*p* < 0.001) and in-hospital mortality rates did not show a statistically significant increase (*p* = 0.271); however, the overall in-hospital mortality rate remained high and increased slightly, reaching 22.5% across the cohort. Independent risk factors for in-hospital mortality included ≥ 65 age, CKD, nosocomial acquisition, *Candida* spp. infection, prosthetic valve IE, vegetation size > 15 mm, and central nervous system emboli (Table 2).

**Table 2:** Variables independently associated with in-hospital mortality of infective endocarditis patients in a multivariable logistic regression model

	Survivor (*n* = 809)	Exitus (*n* = 235)	Univariate analysis *p* value	Multivariate analysis *p* value	OR	%95 CI
Age, years 18–39 y 40–64 y ≥65 y	169(20.9) 414(31.2) 226(27.9)	33(14) 103(43.8) 99(42.1)	Reference < 0.001 < 0.001	Reference 0.692 **0.031**	1.116 1.899	0.648–1.921 1.062–3.399
Chronic kidney disease	152(18.8)	68(28.9)	0.001	**0.003**	1.924	1.257–2.943
Nosocomial	106(13.3)	51(22.1)	0.001	**0.001**	2.114	1.345–3.322
Candida spp.	13(1.6)	14(5.9)	< 0.001	**0.012**	2.875	1.263–6.544
Prosthetic valve IE	225(27.8)	98(41.7)	< 0.001	**0.001**	1.927	1.308–2.838
Vegetation size, > 15 mm	182(22.5)	69(29.4)	0.030	**0.038**	1.551	1.026–2.347
Central nervous system emboli	138(17.1)	65(27.7)	< 0.001	**0.001**	1.978	1.307–2.954

Over the past decade, the proportion of culture-negative IE decreased significantly from 32.7 to 22.4% (*p* = 0.027). Among all patients, the most frequently identified pathogens were staphylococci (36.4%), followed by streptococci (14%) and enterococci (11.9%). A significant increase in staphylococci was observed over the three periods, rising from 27.8 to 41.1% (*p* = 0.007), particularly in *S. aureus*, which increased from 15.6 to 27.8% (*p* = 0.004). No significant change was noted in the frequency of streptococci (*p* = 0.220) (Table 3).

**Table 3:** Microbiological differences between three time periods of overall cohorts

	Total (*n* = 1044)	2013–2016 (*n* = 205)	2017–2020 (*n* = 491)	2021–2023 (*n* = 348)	*p* value
Culture negative IE	281(26.9)	67(32.7)	136(27.7)	78(22.4)	**0.027**
*Staphylococcus* spp.	380(36.4)	57(27.8)	180(36.7)	143(41.1)	**0.007**
*S. aureus* Methicillin resistance	245(23.5) 72(29.4)	32(15.6) 14(43.8)	116(23.6) 29(25)	97(27.9) 29(29.9)	**0.004** 0.185
CoNS Methicillin resistance	135(12.9) 97(71.9)	25(12.2) 24(96)	64(13.1) 45(70.3)	46(13.2) 28(60.9)	0.962 0.052
*Streptococcus* spp.	146(14)	35(17.1)	60(12.2)	51(14.7)	0.220
*Enterococcus* spp.	124(11.9)	21(10.2)	68(13.8)	35(10)	0.129
Enterobacterales	24(2.3)	8(3.9)	14(2.9)	12(3.4)	0.659
Non-fermentatives	18(1.7)	4(2)	11(2.2)	3(0.9)	0.252
*Candida* spp.	27(2.6)	5(2.4)	8(1.6)	14(4)	0.134
*Granulicatella* and *Abiotrophia* spp.	11(1.1)	2(1)	6(1.2)	3(0.9)	0.574
*Coryneobacterium* spp.	10(1)	3(1.5)	5(1)	2(0.6)	0.252
*Coxiella* spp.	9(0.9)	5(2.4)	3(0.6)	2(0.6)	0.688
*Brucella* spp.	7(0.7)	2(1)	1(0.2)	4(1.1)	0.665
*Bartonella spp.*	1(0.1)	-	-	1(0.3)	0.782
HACEK	2(0.2)	1(0.5)	1(0.2)	0(0)	0.594

## Discussion

The epidemiological profile of IE has shifted towards older patients, driven by emerging risk factors [12, 13]. In developed countries, the mean age of IE ranges between 60 and 75 years [5, 13, 14]. Approximately 20 years ago, studies conducted in Türkiye reported a mean patient age of 36 years [15]; however, over the last decade, this has increased to a range of 48.5 to 58.3 years [16, 17, 18, 19]. In the largest previous case series by Şimşek-Yavuz et al. [9], the median age was reported as 47 years, whereas in the current study, it has risen to 57 years, reflecting a consistent upward pattern over the past decade.

The increase in patient age likely reflects changes in IE risk factors. Notably, in the 21st century, rheumatic fever has declined as a predisposing factor, while there has been a significant rise in the number of patients with prosthetic valves, ICDs, and degenerative valvular disease [6]. Similarly, in this study, the most frequently identified risk factors were prosthetic valves, ICDs, and degenerative valve disease. The increasing life expectancy and prevalence of degenerative heart diseases have led to a rise in interventional cardiac procedures, such as ICDs, contributing to the resurgence of CIED-IE [5]. This study demonstrates a statistically significant increase in CIED-IE over the past decade. Conversely, a significant decrease was observed in the prevalence of rheumatic fever and CHD. In the previous largest national case series, rheumatic fever accounted for 33.9% of IE cases [9], while in the present study, this proportion has notably declined to 8.3%. This substantial decrease is highly significant and underscores the changing epidemiology of IE, contributing valuable insights to national data.

Another predisposing risk factor, IVDU, was reported to be 0.9% in previous studies conducted in Türkiye [9], whereas this study found the frequency to be 4.5%. In different studies, the reported frequency has been documented to range between 2.6% and 7.8% [5, 20, 21]. It is well-documented that the rate of IVDU in Türkiye has been increasing annually. According to the Turkey Drug Report 2023, the number of applications to inpatient addiction treatment centers increased from 9,824 in 2020 to 14,042 in 2022 [22, 23]. The rising prevalence of IVDU highlights a significant public health concern and suggests that IVDU may emerge as a critical risk factor for IE in our country as well.

Right-sided IE accounts for approximately 5–10% of all cases, with its lower incidence compared to left-sided IE attributed to fewer pathological conditions affecting right-sided valves, anatomical-vascular differences and lower pressure gradients. The presence of ICDs, IVDU, and central venous catheters can increase the likelihood of right-sided IE, often involving the tricuspid valve [24, 25, 26]. The increase in tricuspid valve involvement to 12.9% during the study period, along with the rise in lead involvement to 9.8% and the prevalence of ICDs to 13.8%, reflects a significant pattern in the Türkiye cohort, highlighting the growing incidence of CIED-IE cases. Additionally, the increasing incidence of pulmonary embolism, one of the most common complication of right-sided IE [24], in this cohort may be associated with the rising prevalence of tricuspid valve involvement and CIED-IE.

In this study, a statistically significant decline in surgical intervention rates was observed, which may be attributed to the changing epidemiological characteristics of the patient cohort. Advanced age, comorbidities, and prior procedures often lead to hesitancy toward surgery among clinicians and patients. Compared to younger patients, it is a well-known fact that surgical intervention is less frequently performed, and mortality rates are higher in elderly infective endocarditis patients [1]. The EURO-ENDO registry demonstrated that surgery was less frequently performed in patients over 80 years of age [27]. A Swedish analysis reported that surgery was underutilized in elderly IE patients, resulting in significantly higher 1-year mortality rates for those who did not undergo surgical intervention [28]. 

Despite advances in medical care, the increasing complexity of patients has contributed to the persistently high mortality rates of IE in recent decades [5]. The literature reports hospital mortality rates for IE ranging from 15 to 30% [6, 22, 30]. Previous studies conducted in Türkiye have reported higher hospital mortality rates, ranging from 27.8 to 36% [9, 17, 19]. In this study, the hospital mortality rate was 22.8%, reflecting a decline compared to earlier national studies; however, it remains alarmingly high.

Numerous studies have identified key risk factors associated with in-hospital mortality in IE, including ≥ 65 age, *S. aureus* or fungal etiology, prosthetic valve, dual-valve involvement, neurological complications, and CKD requiring HD [29, 30, 31, 32]. In particular, HCA-IE infections in prosthetic valve endocarditis have been recognized as a significant contributor to increased mortality [10]. Consistent with the literature, this study identified older age, CKD, nosocomial infection, candidal etiology, prosthetic valve involvement and CNS embolism as significant risk factors associated with increased mortality in IE. Previous studies have demonstrated that a vegetation size greater than 10 mm is associated with an increased risk of embolic complications and mortality [33]. However, in this study, the threshold value was identified as 15 mm. Similarly, studies conducted by Erbay et al. [34] and Thurny et al. [35] also reported that vegetations larger than 15 mm were significant risk factors for mortality.

Changes in the at-risk population for IE have significantly influenced its microbiological profile [31]. Chronological increases in the frequency of *S. aureus* IE over recent decades are well-documented in the literature [7]. Consistent with these findings, our study identified *Staphylococcus* spp. as the most frequently observed pathogens in all type IE, with a particularly notable rise in *S. aureus* cases over the study period. This predominance of *S. aureus* is likely associated with increasing patient age, the rising prevalence of CIED-IE, HCA-IE, and IVDU. These changes in the etiological microorganism profile can influence the clinical presentation and complication patterns of IE. Spondylodiscitis is the most common osteoarticular complication of IE [10], and its increasing incidence has been documented in this study. *S. aureus* is well recognized as the predominant causative pathogen of spondylodiscitis [36], and its increasing prevalence in this cohort is likely linked to the rising incidence of this complication observed in this study.

The decreasing prevalence of culture-negative IE in our country is very important. The establishment of proper blood culture collection practices before initiating antibiotics, along with the diagnostic algorithms outlined in the Turkey Consensus Report [37], have contributed to a reduction in the rate of culture-negative endocarditis. In 0.9% of culture-negative endocarditis cases, *Coxiella* was identified as the causative agent, while *Brucella* accounted for 0.7% and *Bartonella* for 0.1%. One of the focal complications of brucellosis, which is endemic in our country, is endocarditis [38, 39]. In a study conducted by Şimşek et al. in our country [9], brucellosis was identified as the cause of infective endocarditis in 4.6% of cases, whereas in our study, this rate was found to be 0.7%, which is surprising.

The limitations of this study can be summarized as follows. Firstly, this was a retrospective study. Although the primary centers for the diagnosis and treatment of infective endocarditis remained constant throughout the study period, the incorporation of additional centers over time may have introduced potential bias. The cases in this study were classified as possible or definite IE according to the 2015 ESC criteria, which were valid at the time. However, as diagnostic criteria continue to evolve, the potential for overdiagnosis or missed cases remains a limitation of this study. While a significant part of the culture-negative endocarditis cases is attributed to antibiotic use, this information could not presented in this study. Furthermore, due to insufficient data, the effects of endocarditis prophylaxis on epidemiological outcomes could not be investigated. The lack of the examination of treatment modalities and surgery-related data in detail is another limitation of the study. The decline in case numbers during the third time period may be attributed to the impact of the COVID-19 pandemic on case reporting processes, as data reporting from major cardiac centers remained consistent, while some centers experienced disruptions in data flow, potentially introducing a source of bias. Generalisability is limited despite its large sample size and country-wide-distributed setting. Providing a nationwide perspective on infective endocarditis, addressing certain knowledge gaps in this field, and integrating clinical and microbiological findings to interpret national data constitute major strengths of this study.

The epidemiology of IE is evolving due to several factors leading to changes in its microbiological characteristics and clinical presentation. Patients are now generally older and have a greater burden of comorbidities. Prosthetic valves, ICDs, and HD have emerged as the risk factors, replacing rheumatic disease and CHD. *Staphylococcus* spp. has become the most commonly isolated pathogen. The management of IE patients should be guided by clinical guidelines, with consideration of up-to-date epidemiological data to ensure comprehensive and evidence-based care.

## Electronic supplementary material

Below is the link to the electronic supplementary material.

Supplementary Material 1

## Data Availability

The datasets generated during and/or analysed during the current study are available in the Elif M. Saricaoglu repository. Data are available in the hospital’s electronic medical records.
