# Supplementary material for: Epidemiological, clinical and microbiological aspects of infective endocarditis in Türkiye
Source: Eur J Clin Microbiol Infect Dis. 2025 Mar 14;44(6):1325–33. doi: 10.1007/s10096-025-05095-8 (PMC12116849; doi:10.1007/s10096-025-05095-8)
Supplement: Supplementary file 1 — Supplementary Material 1 [file 10096_2025_5095_MOESM1_ESM.docx]

|  | 2013-2016  (n=205) | 2017-2020  (n=491) | 2021-2023  (n=348) | Toplam  (n=1044) | p değeri |
| --- | --- | --- | --- | --- | --- |
| **Demographics** | | | | | |
| Age | 54 (40,65) | 55(43,66) | 60(47,70) | 57(44,68) | **<0.001** |
| <65 years  ≥65 years | 152(74.1)  53(25.9) | 354(72.1)  137(27.9) | 213(61.2)  135(38.8) | 719(68.9)  325(31.1) | **0.001** |
| Male gender | 133(64.9) | 310(63.1) | 223(64.1) | 666(63.8) | 0.901 |
| **Comorbidities** |  |  |  |  |  |
| Hypertension | 66(32.2) | 157(32) | 159(45.7) | 382(36.6) | **<0.001** |
| Diabetes mellitus | 39(19) | 114(23.2) | 129(37.1) | 282(27) | **<0.001** |
| Chronic kidney disease | 40(19.5) | 97(19.8) | 83(23.9) | 220(21.1) | 0.297 |
| Coronary artery disease | 40(19.5) | 104(21.2) | 75(21.6) | 219(21) | 0.841 |
| Congestive heart failure | 21(10.2) | 44(9) | 55(15.8) | 120(11.5) | **0.008** |
| Chronic obstructive pulmonary disease | 16(7.8) | 39(7.9) | 36(10.3) | 91(8.7) | 0.418 |
| Malignancy | 9(4.4) | 19(3.9) | 29(8.3) | 57(5.5) | **0.015** |
| Autoimmun disease | 3(1.5) | 23(4.7) | 18(5.2) | 44(4.2) | 0.086 |
| Cirrhosis | 1(0.5) | 7(1.4) | 6(1.7) | 14(1.3) | 0.463 |
| **Predisposition conditions** | | | | | |
| Prosthetic valve | 59(28.8) | 166(33.8) | 98(28.2) | 323(30.9) | 0.166 |
| Degenerative heart disease | 48(23.4) | 89(18.1) | 46(13.2) | 183(17.5) | **0.009** |
| Hemodialysis | 27(13.2) | 84(17.1) | 58(16.7) | 169(16.2) | 0.419 |
| Intracardiac device | 15(7.3) | 34(6.9) | 53(15.2) | 102(9.8) | **<0.001** |
| Rheumatic fever | 23(11.2) | 39(7.9) | 25(7.2) | 87(8.3) | 0.230 |
| Bicuspid aortic valve | 16(7.8) | 35(7.1) | 13(3.7) | 64(6.1) | 0.070 |
| Congenital heart disease | 17(8.3) | 32(6.5) | 11(3.2) | 60(5.7) | **0.026** |
| Previous IE episode | 14(6.8) | 22(4.5) | 16(4.6) | 52(5) | 0.397 |
| Intravenous drug user | 6(2.9) | 28(5.7) | 13(3.7) | 47(4.5) | 0.192 |
| **Symptoms and clinical findings** | | | | | |
| Duration of symptoms, days | 15(7,32) | 15(7,30) | 12(5,30) | 14(7,30) | 0.169 |
| Fever | 167(81.5) | 398(81.1) | 249(71.6) | 814(78) | **0.002** |
| Fatique | 89(43.4) | 278(56.6) | 239(68.7) | 606(58) | **<0.001** |
| Dyspnea | 57(27.8) | 135(27.5) | 114(32.8) | 306(29.3) | 0.228 |
| Muscle-joint pain | 29(14.1) | 80(16.3) | 74(21.3) | 183(17.5) | 0.064 |
| Altered consciousness | 23(11.2) | 72(14.7) | 55(15.8) | 150(14.4) | 0.321 |
| Weight loss | 14(6.8) | 69(14.1) | 56(16.1) | 139(13.3) | 0.007 |
| Murmur | 96(59.6) | 253(54.2) | 153(44) | 501(48.1) | **0.001** |
| Palpitation | 17(8.3) | 44(9) | 49(14.1) | 110(10.5) | **0.03** |
| Rash* | 7(3.4) | 20(4.1) | 13(3.7) | 86(8.2) | 0.912 |
| Osler’s nodes | 5(2.7) | 16(3.6) | 3(0.9) | 24(2.3) | 0.080 |
| Janeway lesions | 3(1.6) | 10(2.2) | 9(2.6) | 22(2.1) | 0.778 |
| Splinter hemorrhages | 3(1.6) | 10(2.2) | 14(4) | 27(2.6) | 0.188 |
| Roth spot | 4(4.3) | 11(3.5) | 7(3.8) | 22(2.1) | 0.938 |
| Hematuria | 275(47.6) | 181(45.5) |  | 456(43.7) | 0.518 |
| Proteinuria | 219(37.8) | 184(46.2) |  | 403(38.6) | **0.009** |
| **Site of infection acquisition** | | | | | |
| Community-acquired | 133(64.9) | 269(54.8) | 217(62.4) | 619(59.3) | 0.709 |
| Nosocomial | 29(14.1) | 72(14.7) | 56(16.1) | 157(15) | **0.010** |
| Non-nosocomial healthcare-associated infection | 43(21) | 150(30.5) | 75(21.6) | 268(25.7) | 0.484 |
| **Endocarditis side** | | | | | |
| Mitral | 90(43.9) | 217(44.2) | 162(46.6) | 469(44.9) | 0.510 |
| Aortic | 102(49.8) | 203(41.3) | 97(27.9) | 402(38.5) | **<0.001** |
| Trikuspid | 15(7.3) | 51(10.4) | 45(12.9) | 111(10.6) | **0.035** |
| Pulmonary | 3(1.5) | 7(1.4) | 3(0.1) | 13(1,3) | 0.561 |
| Lead | 8(3.9) | 28(5.7) | 34(9.8) | 70(6.7) | **0.011** |
| Endocardium | 4(2) | 8(1.6) | 9(2.6) | 21(2) | 0.189 |
| **Type of IE** | | | | | |
| Native valve IE | 136(66.3) | 302(61.5) | 221(63.2) | 659(63) | 0.482 |
| Prosthetic valve IE | 58(28.3) | 156(31.8) | 93(26.7) | 307(29.4) | 0.266 |
| Cardiac implantable electronic device-associated IE | 11(5.4) | 32(6.5) | 35(10.1) | 78(7.5) | 0.070 |
| **Echocardiographic findings** | | | | | |
| Vegetation | 183(89.3) | 423(86.2) | 285(81.9) | 891(85.3) | **0.048** |
| Vegetation size |  |  |  |  | 0.272 |
| 0-10 mm | 50(36.8) | 153(39.9) | 117(42.7) | 320(30.7) |  |
| 11-15 mm | 34(25) | 106(27.7) | 82(29.9) | 222(21.3) |  |
| 15 mm üzeri | 52(38.2) | 124(32.4) | 75(27.4) | 251(24) |  |
| Perivalvular abscess | 12(5.9) | 25(5.1) | 16(4.6) | 53(5.1) | 0.809 |
| Prosthetic valve dehiscence, fistula, aneurysm | 8(3.9) | 26(5.3) | 16(4.6) | 50(4.8) | 0.720 |
| Chordal rupture | 1(0.5) | 7(1.4) | 0(0) | 8(0.8) | 0.058 |
| New-onset valve regurgitation | 1(0.5) | 14(2.8) | 24(6.9) | 39(3.7) | **<0.001** |
| **Microbiological findings** | | | | | |
| Blood culture negative | 67(32.7) | 136(27.7) | 78(22.4) | 281(26.9) | **0.027** |
| *Staphylococcus* spp. | 57(27.8) | 180(36.7) | 143(41.1) | 380(36.4) | **0.007** |
| *S. aureus* | 32(15.6) | 116(23.6) | 97(27.9) | 245(23.5) | **0.004** |
| Methicillin resistance | 14(6.8) | 29(5.9) | 29(8.3) | 72(6.8) | 0.185 |
| CoNS  Methicillin resistance | 25(12.2)  24(11.7) | 64(13.1)  45(9.2) | 46(13.2)  28(8) | 135(12.9)  97(9.4) | 0.962  **0.042** |
| *Streptococcus* spp. | 35(17.1) | 60(12.2) | 51(14.7) | 146(14) | 0.220 |
| *Enterococcus* spp. | 21(10.2) | 68(13.8) | 35(10) | 124(11.9) | 0.129 |
| *Enterobacterales* | 8(3.9) | 14(2.9) | 12(3.4) | 24(2.3) | 0.659 |
| Gram negative non-fermentative | 4(2) | 11(2.2) | 3(0.9) | 18(1.7) | 0.252 |
| *Candida* spp. | 5(2.4) | 8(1.6) | 14(4) | 27(2.6) | 0.134 |
| *Brucella* spp. | 2(1) | 1(0.2) | 4(1.1) | 7(0.7) | 0.665 |
| HACEK | 1(0.5) | 1(0.2) | 0(0) | 2(0.2) | 0.594 |
| *Granulicatella* and *Abiotrophia* spp. | 2(1) | 6(1.2) | 3(0.9) | 11(1.1) | 0.574 |
| *Coryneobacterium* spp. | 3(1.5) | 5(1) | 2(0.6) | 10(1) | 0.252 |
| **Complications** |  |  |  |  |  |
| Cranial embolism | 37(18) | 106(21.6) | 60(17.2) | 203(19.4) | 0.250 |
| Spleen abscess | 2(1) | 19(3.9) | 2(0.6) | 23(22.2) | 0.353 |
| Glomerulonephritis | 1(0.5) | 21(4.3) | 7(2) | 29(2.8) | 0.593 |
| Pulmonary thromboembolism | 2(1) | 32(6.5) | 43(12.4) | 77(7.4) | **<0.001** |
| Spondylodiscitis | 1(0.5) | 7(1.4) | 16(4.6) | 24(2.3) | **0.002** |
| Heart failure | 10(4.9) | 52(10.6) | 58(16.7) | 120(11.5) | **<0.001** |
| Cardiac surgery for IE | 102(49.8) | 196(39.9) | 108(31.1) | 406(38.8) | **<0.001** |
| In-hospital mortality | 38(18.5) | 112(22.8) | 85(24.4) | 235(22.5) | 0.271 |

*The results were reported as medians and Quartile 1–Quartile 3 (Q1–Q3) for non-normally distributed and ordinal variables, and frequencies for categorical variables
